# Overcoming barriers to providing comprehensive inpatient care during the COVID-19 pandemic

**DOI:** 10.6061/clinics/2020/e2100

**Published:** 2020-06-24

**Authors:** Anna Miethke-Morais, Beatriz Perondi, Leila Harima, Amanda C. Montal, Renato Madrid Baldassare, Danielle P. Moraes, Lucila Pedroso, Marcelo C.A. Ramos, Solange R.G. Fusco, Antonio José Pereira, Tarcísio E.P. Barros-Filho, Eloisa Bonfá, Edivaldo M. Utiyama, Aluisio C. Segurado

**Affiliations:** Hospital das Clinicas HCFMUSP, Faculdade de Medicina, Universidade de Sao Paulo, Sao Paulo, SP, BR

Hospital das Clínicas (HCFMUSP), affiliated to the Academic Health Center of the Faculty of Medicine, University of São Paulo, is the largest public hospital complex in Latin America, with 2400 beds. This center comprises eight specialized institutes, including the Central Institute (ICHC), an eleven-floor multidisciplinary tertiary care facility with 900 beds, of which, 90 are in intensive care units (ICUs).

The rapid progression of the coronavirus disease (COVID-19) pandemic in São Paulo and the consequent need to adapt hospital care provision to this public health emergency led the institutional Crisis Management Committee (CMC) to designate the Central Institute as a dedicated unit for COVID-19 care, which was accomplished in 8 days. The subsequent challenge was to repurpose wards that were formerly set up as specialized inpatient care units, with 500 ward beds, into wards solely devoted to COVID-19 care.

The ICHC includes medical staff from 33 different medical specialties. To maximize the quality of inpatient care to COVID-19 patients, clinicians and anesthesiologists with appropriate skills to manage critically ill patients were allocated to the ICUs and emergency department. The remaining highly specialized medical staff (rheumatologists, endocrinologists, liver transplantation surgeons, urologists, and orthopedists, among others) were designated to care for patients in 20 regular wards, alongside medical residents, also recruited from different specialties. Although motivated to take part in the institutional COVID-19 response, the lack of previous consolidated experience in emergency care, expectedly, triggered fear and discomfort among the medical staff. In fact, for those not familiar with the field, managing acute respiratory distress syndrome, which frequently required intubation and mechanical ventilation, was unnerving (1). This issue was immediately identified, and after listening and staying connected to the teams on a day-to-day basis (2), the CMC was able to implement different measures to help improve their well-being and adherence to the hospital’s response model.

## Personal safety - Decreasing the fear of infection

Health professionals’ safety is a priority during disasters, particularly when dealing with a contagious disease. Managing COVID-19 patients requires continuous provision of sufficient personal protective equipment (PPE), along with establishment of clear rules and guidelines on where, when, and how to use PPE, as well as its correct donning and doffing (3). These aspects were managed by sizing and stocking PPE in advance, improving the logistics of PPE distribution to wards, maintaining written PPE-use protocols, and providing structured donning and doffing training with practical simulation.

## Team organization, training, and support - Overcoming the discomfort associated with the lack of previous experience in the field

After years of experience in their particular medical field, doctors had to treat patients with a condition that is very different from that seen in their daily practice. To overcome the distress associated with this, we developed a comprehensive team organization, training, and support plan that included:

1. A standardized treatment protocol: This was designed by specialists (infectious disease specialists and pulmonologists) and was included as part of the training program for the medical staff and residents before they were designated to treat COVID-19 patients. Practical guidelines and videos (when and how to request for severe acute respiratory syndrome coronavirus 2 (SARS-CoV-2) testing and clear hospital discharge criteria, among others) were made available on the hospital’s website.
2. Organizing a ward medical “team”: A ward medical “team” consisted of a leader, who was a specialist, and 12 residents for every 25 beds. The specialist leaders remained in their usual ward, aiming to provide care in a more familiar setting. Their working hours were adjusted so that at least one of the physicians would be on duty in daily shifts (7 a.m. to 7 p.m.). Medical residents, from different specializations, remained in the same ward for at least one month, working in 12-hour shifts followed by 36 hours of rest; four of them worked during the day shift and two, in the night shift.
3. 24-hour specialist support in wards: An expert (infectious disease, pulmonology, or internal medicine specialist) was available as a focal supervisor to the ward medical team at all times to provide counseling on patient management. During the day, an expert was available for every two wards, and at night, for every three to four wards.
4. Back up if the patients’ clinical condition worsened: A rapid response team, consisting of anesthesiologists with airway management expertise, trained in COVID-19 airway management protocols, and equipped with difficult airway tools, was made available 24/7, responding in less than 3 minutes when called to any hospital area where intubation and mechanical ventilation were required. Not having to cope with this stressful scenario alone (4) relieved the medical staff and strengthened their motivation toward being a part of and leading ward care teams. Rapid and preferential transfer of patients in a critical condition to the ICU beds in operation at the Central Institute (300 beds) was also undertaken by a transport team.

## Caring for clinicians’ mental health - Coping with COVID-19

Anxiety, burnout, and other mental health conditions are frequent among healthcare workers aiding in disaster scenarios (4). Therefore, it is essential to implement strategies to safeguard clinicians’ mental health in these scenarios. Thus, from the initiation of the hospital’s response to COVID-19, a team of mental health providers (psychiatrists and psychologists) was made readily available to support every healthcare professional or anyone working in the hospital. They could be accessed through a 24/7 hotline or scheduled in-person care appointments, whenever necessary.

In conclusion, important lessons were learned in organizing COVID-19 inpatient care at Hospital das Clinicas in the context of a public health emergency. Staying in close contact with first responders on a day-to-day basis to ask about their specific needs and to listen and acknowledge their requests helped strengthen staff collaboration. Moreover, it was crucial to identify the need to implement strategies focused on mitigating some of the clinicians’ main sources of anxiety and discomfort. This allowed proper functioning of hospital wards, with participation of all available medical staff in HCFMUSP’s response to COVID-19.

## AUTHOR CONTRIBUTIONS

All coauthors have provided substantial intellectual contributions to the manuscript and agree with its content and submission. The manuscript has not been previously published and is not under consideration for publication in any journal.
